# Theoretical proposal for restoration of hand motor function based on plasticity of motor-cortical interhemispheric interaction and its developmental rule

**DOI:** 10.3389/fneur.2024.1408324

**Published:** 2024-07-23

**Authors:** Hideki Nakano, Yandi Tang, Tomoyo Morita, Eiichi Naito

**Affiliations:** ^1^Center for Information and Neural Networks (CiNet), Advanced ICT Research Institute, National Institute of Information and Communications Technology (NICT), Osaka, Japan; ^2^Faculty of Health Sciences, Kyoto Tachibana University, Kyoto, Japan; ^3^Graduate School of Frontier Biosciences, Osaka University, Osaka, Japan

**Keywords:** motor cortex, interhemispheric interaction, fMRI, EMG, neurorehabilitation, development, hand motor function, proprioception

## Abstract

After stroke, the poorer recovery of motor function of upper extremities compared to other body parts is a longstanding problem. Based on our recent functional MRI evidence on healthy volunteers, this perspective paper proposes systematic hand motor rehabilitation utilizing the plasticity of interhemispheric interaction between motor cortices and following its developmental rule. We first discuss the effectiveness of proprioceptive intervention on the paralyzed (immobile) hand synchronized with voluntary movement of the intact hand to induce muscle activity in the paretic hand. In healthy participants, we show that this bilateral proprioceptive-motor coupling intervention activates the bilateral motor cortices (= bilaterally active mode), facilitates interhemispheric motor-cortical functional connectivity, and augments muscle activity of the passively-moved hand. Next, we propose training both hands to perform different movements, which would be effective for stroke patients who becomes able to manage to move the paretic hand. This bilaterally different movement training may guide the motor cortices into left–right independent mode to improve interhemispheric inhibition and hand dexterity, because we have shown in healthy older adults that this training reactivates motor-cortical interhemispheric inhibition (= left–right independent mode) declined with age, and can improve hand dexterity. Transition of both motor cortices from the bilaterally active mode to the left–right independent mode is a developmental rule of hand motor function and a common feature of motor function recovery after stroke. Hence, incorporating the brain’s inherent capacity for spontaneous recovery and adhering to developmental principles may be crucial considerations in designing effective rehabilitation strategies.

## 1 Introduction

The poorer recovery of motor function of upper extremities after stroke compared to other body parts is a longstanding problem (1). This perspective paper proposes systematic hand motor rehabilitation utilizing the plasticity of interhemispheric interaction between the motor cortices and following its developmental rule, based on recent neuroscientific evidence.

### 1.1 Motor-cortical activity after stroke

The motor cortex (precentral gyrus), which includes the primary motor cortex (M1) and dorsal premotor cortex (PMD), is the executive locus of motor control; damage to this area or descending tracts from this area can cause severe motor paralysis of the limbs. However, brain plasticity allows for functional recovery even in adult brains (2, 3). Interestingly, this functional recovery does not occur randomly. For example, when a patient manages to move a paretic hand after stroke, the contralesional motor cortex ipsilateral to the hand is often recruited in addition to the ipsilesional one, so that bilateral activity can be observed (2, 4). This bilaterally active mode is a spontaneous brain reaction observed relatively early after stroke (within 10 days), and can be considered a state whereby the brain is searching for a new motor control pathway (including a pathway from the ipsilateral motor cortex) by trial and error to move the paretic hand. In other words, this bilateral mode is the first step toward restoring motor function (2). Recent animal studies have suggested that this bilaterally active mode is caused by disinhibition of interhemispheric inhibition between the left and right motor cortices (5) by acetylcholine modulating GABAergic interneurons (6).

### 1.2 Motor-cortical activity in healthy adults

In the brain of typically developed young adults, there are interhemispheric facilitatory and inhibitory circuits between the two motor cortices (7). When young adults perform simple unilateral movements (e.g., simple button pressing with a finger or simple hand alternating extension-flexion movements), the contralateral motor cortex is usually activated, while the ipsilateral motor cortex is inhibited (8–11), probably due to interhemispheric inhibition between the two motor cortices (12). On the other hand, when young adults perform complex unilateral movements (e.g., stick-spinning or ball rotation with multiple fingers), there is activity in the ipsilateral motor cortex (especially in the PMD) in addition to the contralateral activity (8, 13, 14). Thus, the human brain adaptively controls movement by flexibly and plastically altering interhemispheric inhibition between the two motor cortices.

### 1.3 Developmental of motor-cortical interhemispheric inhibition

The interhemispheric inhibition is not innate. During childhood, interhemispheric inhibition between the left and right motor cortices is still immature, maturing during adolescence (9, 10, 15). Hence, the motor cortex before adolescence is in a bilaterally active mode, but this begins to change in adolescence to a left–right independent mode that allows the left and right hands to move independently. On the other hand, interhemispheric interaction between the two motor cortices can be greatly affected by training. We have recently shown that a top wheelchair racing Paralympian who trained from school age for special training in wheelchair racing, which requires bimanually synchronized upper-limb movements, shows a bilaterally active mode even in adulthood. She showed bilateral motor-cortical activations even during a simple alternating extension-flexion movement of the right hand, which should be called hyper-adaptation phenomenon rarely seen in typically-developed people (11). This finding inspired an intervention using bimanually synchronized movements that can act on the plasticity of interhemispheric interaction between the two motor cortices.

In this paper, we first propose the effectiveness of an intervention to facilitate the motor cortices into bilaterally active mode, i.e., passive movement of one hand synchronized with voluntary movement of the other hand, based on our recent findings in healthy younger adults (Section 2). Next, we propose the effectiveness of training both hands to perform different movements to guide the motor cortices into left–right independent mode, thereby improving interhemispheric inhibition and hand dexterity, based on our previous findings on healthy older adults (Section 3). By doing so, this paper provides a theoretical and systematic framework for the interventions that utilize the higher plasticity of motor-cortical interhemispheric interaction and that follow its developmental rule.

## 2 Induction of muscle activity utilizing bilaterally active mode

**Figure 1:**
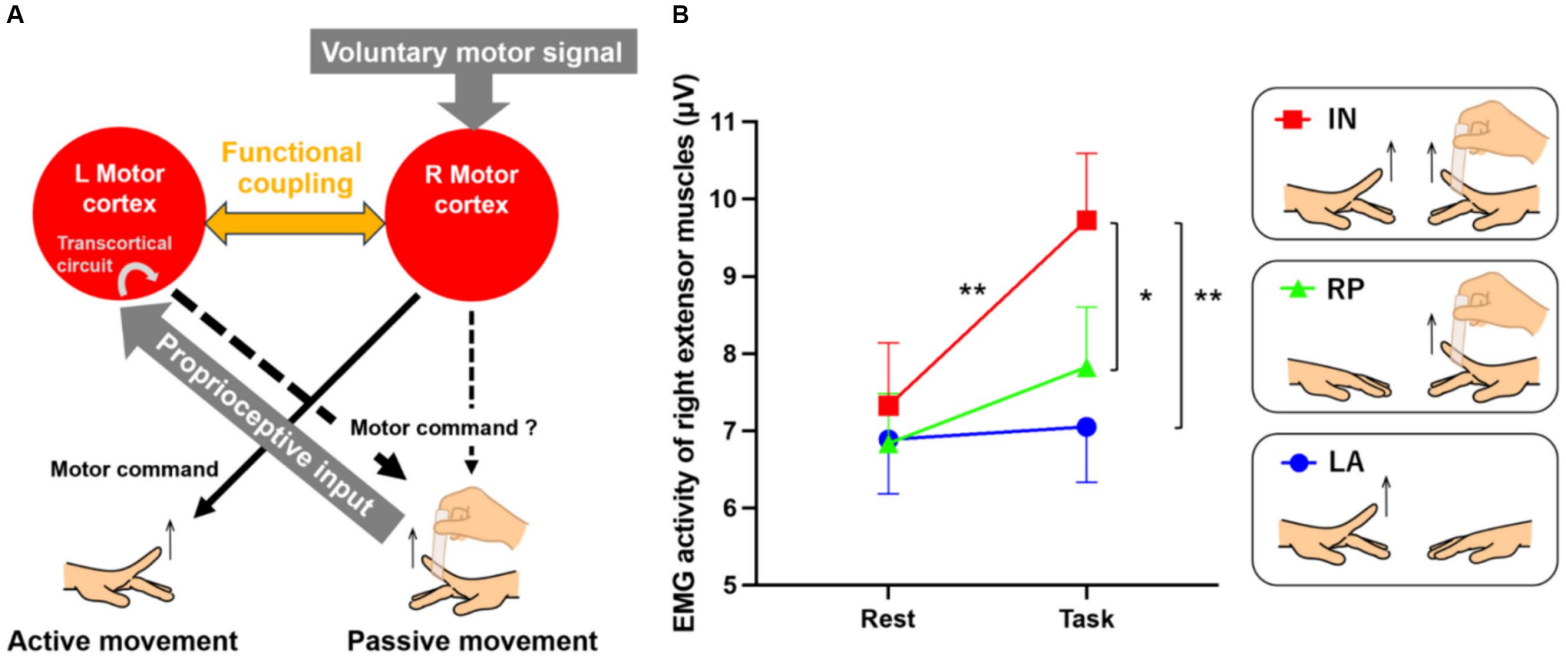
Possible neural mechanisms underlying passive extension of the right index finger synchronized with the voluntary extension of the left finger **(A)** and EMG results **(B)**. **(A)** We hypothesize that passive movement of the right hand synchronized with voluntary movement of the left hand will lead the left and right motor cortices to a bilaterally active mode, and interhemispheric functional connectivity must be enhanced between the two motor cortices. This enhanced functional coupling may allow the bilateral motor cortices to share the voluntary motor signal input to the right motor cortex and the proprioceptive input to the left motor cortex, promoting sensory-motor associations between them. If the sensory-motor association occurs in the left motor cortex, the voluntary motor signal may effectively activate the intrinsic transcortical circuit between the left motor cortex and the right hand muscles, leading to muscle activity increase in the right passively-moved finger. If this association also occurs in the right motor cortex, the cortex might also become part of the network increasing the muscle activity, in concert with the left one. **(B)** EMG results from the left active (LA; blue), right passive (RP; green), and in-phase (IN; red) tasks. Small bars on the graph indicate standard errors of the mean across participants. ^*^p < 0.005, ^**^p < 0.001.

To move a paralyzed hand, it is first necessary to allow muscle activity in the paretic hand to emerge. Here, we consider an intervention that maximizes the bilaterally active mode, possibly the first step in the restoration of motor function after stroke. In the case of a hemiplegic patient who cannot move the right hand but can move the left hand, we propose a method in which his/her right hand is moved passively synchronized with voluntary movement of the left hand (Figure 1A).

Recently, an intervention to restore hand motor function in hemiplegic patients by electrically contracting the muscles of the paralyzed hand in accordance with the movement of the intact hand [= contralaterally controlled functional electrical stimulation (CCFES)] has been shown to be effective (16–22). One may assume that the essence in this intervention is proprioceptive intervention of a hand synchronized with voluntary movement of the other hand (bilateral proprioceptive-motor coupling). Cunningham et al. (16) have suggested in chronic stroke patients that the CCFES may induce disinhibition of motor-cortical interhemispheric inhibition. However, changes in motor-cortical activity by a bilateral proprioceptive-motor coupling intervention remain unclear. Therefore, we first show in healthy volunteers that passive movement of one hand synchronized with voluntary movement of the other hand can effectively induce muscle activity in the former, and its related activity change in the bilateral motor cortices.

When a healthy person voluntarily moves the left hand, the right motor cortex is activated, and the left motor cortex is deactivated (inhibited) due to interhemispheric inhibition. Similarly, when the right hand is moved passively, a proprioceptive signal activates the left motor cortex via somatosensory area 3a and the cerebellar vermis (23), leading to inhibition of the right motor cortex through interhemispheric inhibition (24).

We hypothesize that simultaneous voluntary movement of the left hand and passive movement of the right hand will lead the left and right motor cortices to a bilaterally active mode (Figure 1A). In this situation, interhemispheric functional connectivity must be enhanced between the motor cortices. This enhanced functional coupling may allow the bilateral motor cortices to share the voluntary motor signal input to the right motor cortex and the proprioceptive input to the left motor cortex, promoting sensory-motor associations between them. If the sensory-motor association occurs in the left motor cortex, the voluntary motor signal may effectively activate the intrinsic transcortical circuit between the left motor cortex and the right hand muscles, for instance (25, 26), increasing muscle activity during passive movement. If this association also occurs in the right motor cortex, the cortex might become part of the network increasing muscle activity, in concert with the left one.

### 2.1 Methods and results

We tested these hypotheses in healthy adults. The details of methods are described in Supplementary material. We recruited 55 healthy right-handed young adults (37 male, 18 female; age 19–26 years old). Their handedness was assessed by the Edinburgh Handedness Inventory (27). The motor tasks consisted of (1) a left finger active extension task (left active; LA) in which the blindfolded participants extended their left index finger to a 1 Hz tone, (2) a right finger passive extension task (right passive; RP) in which the experimenter extended the right relaxed index finger to a 1 Hz tone, and (3) an in-phase task (in-phase; IN) in which the experimenter extended the right relaxed finger (RP) synchronized with the participant’s active left finger extension (LA; Figure 1B). The study protocol was approved by the Ethics Committee of the National Institute of Information and Communications Technology, and the MRI Safety Committee of the Center for Information and Neural Networks (CiNet; no. 2003260010). We explained the details of the present study to all participants before the experiment, and they then provided written informed consent. The study was conducted according to the principles and guidelines of the Declaration of Helsinki (1975).

We first examined if muscle activity in the right relaxed finger increases during the IN task. In all tasks, surface electromyograms (EMGs) were recorded from the finger extensor muscles of the right hand. The 20-s task was repeated eight times with a 10-s rest phase in between. The root-mean-square EMG values from the first 2–19 s during the task and from the first 2–7 s during the rest phase were calculated, and average values for the eight tasks and rest phases were calculated for each individual. A total of 44 of all participants showed EMG increase during the task phase compared to the rest phase in the IN task. A two-factorial analysis of variance (repeated measurement) for tasks (3) × period (2: task-rest) revealed a significant interaction [*F*(2,108) = 9.21; *p* < 0.001]. A *post hoc* test revealed a significant increase in muscle activity during the IN task compared to the rest phase (*p* < 0.001 Bonferroni corrected); further, activity during the IN task was significantly higher than during the LA and RP tasks (*p* < 0.001, *p* < 0.005 Bonferroni corrected, respectively). In other words, the IN task could effectively increase muscle activity in the right relaxed finger, an effect that could not be induced by passive movements alone (Figure 1B).

To investigate the neural substrates underlying the IN task, brain activity was measured with a 3 Tesla functional MRI during the above three tasks. Using an image analysis software of statistical parametric mapping, we preprocessed individual images, spatially smoothed them with a 4 mm Gaussian filter, and conducted statistical analyses (see Supplementary material). We adopted a voxel-wise threshold of *p* < 0.005, and evaluated the significance of brain activations in terms of the spatial extent of the activations in the entire brain or in small volume correction [SVC; *p* < 0.05, family-wise-error (FWE) corrected].

**Figure 2:**
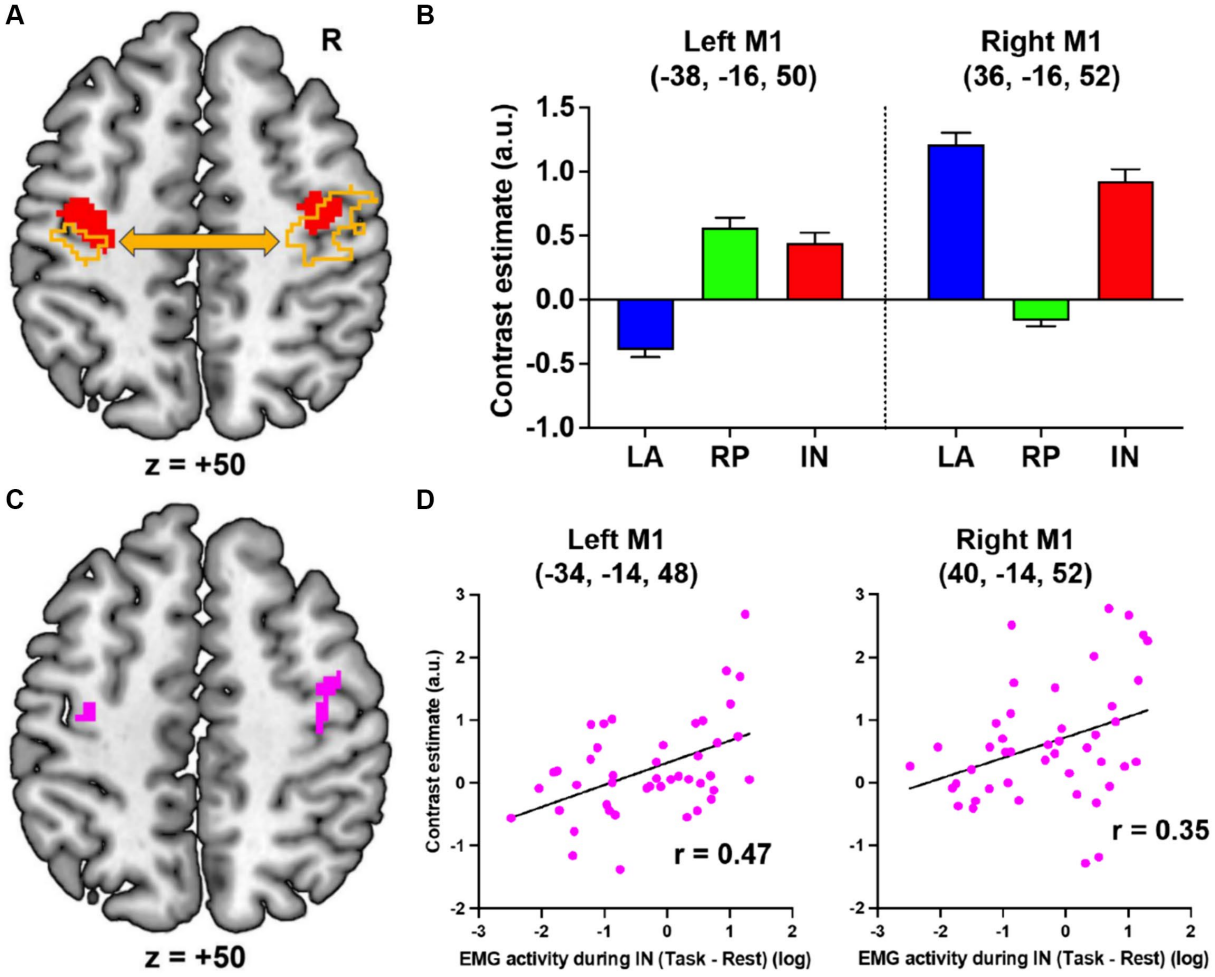
fMRI results. **(A)** Bilateral motor-cortical activations (red) when comparing the IN task with the LA and RP tasks. The left and right motor cortices (orange) in which activity enhanced functional coupling with the right or left motor-cortical cluster (red), respectively. **(B)** Brain activity in the peaks of the left and right motor-cortical clusters in each task. **(C)** The left and right motor cortices (pink) in which activity significantly correlated with the EMG activity during the IN task. **(D)** Interparticipant correlation between the left or right M1 activity and log value of EMG activity. LA, left active; RP, right passive; IN, in-phase; M1, primary motor cortex; R, right; a.u., arbitrary unit.

When comparing the IN task with the LA and RP tasks, bilateral motor-cortical activations were observed (Figure 2A, red). The peaks of these activations were located in M1 (cytoarchitectonic areas 4a/4p; MNI coordinates x, y, z = −38, −16, 50 and 36, −16, 52). Specifically, in the LA task, the right M1 was activated and the left M1 was deactivated while, in the RP task, the opposite was observed (Figure 2B). Hence, when performing the LA or RP task alone, there is deactivation by interhemispheric inhibition, however the IN task, which combines the LA and RP tasks, bilaterally activates motor cortices (Figures 2A,B). Since these activations were observed only during the IN task, these can be considered IN task-related motor-cortical activations, and are likely associated with sensory-motor association.

Next, using a generalized psychophysiological interaction analysis (28), we further examined the brain regions where activity enhanced functional coupling with the activity in the left or right motor-cortical cluster (Figure 2A, red) during the IN task when compared to the other tasks (see Supplementary material). The right (2,202 voxels; peak coordinates = 34, −18, 50, area 4p) or left (746 voxels; peak coordinates = −38, −20, 52, area 4p) motor-cortical activity increased their functional coupling with the left or right cluster, respectively (Figure 2A, orange).

Finally, we examined if the IN task-related motor-cortical activity (IN > LA + RP) correlates with the EMG increase (task > rest) in the right extensor muscles during the IN task across the 44 participants (see Supplementary material). In the left motor cortex, we found a significant cluster of 28 voxels (peak coordinates = −34, −14, 48; Figure 2C, left pink section), which became significant after SVC (*p* = 0.021 FWE-corrected) within a sphere with 8 mm radius around the peak of the IN task-related left M1 activity. Plotting this relationship across participants showed that the IN task-related left M1 activity was greater in participants with higher EMG activity during the IN task (r = 0.47; Figure 2D, left). Similarly, in the right motor cortex, we also found a significant cluster of 27 voxels (peak coordinates = 40, −14, 52; Figure 2C, right pink section), which became significant after SVC (*p* = 0.023 FWE-corrected) within a sphere with 8 mm radius around the peak of the IN task-related right M1 activity. The IN task-related right M1 activity was also greater in participants with higher EMG activity during the IN task (r = 0.35; Figure 2D, right). The results indicate that the IN task-related bilateral motor-cortical activities are related to the EMG increase during the IN task.

### 2.2 Discussion and possible clinical application

The present study has clearly demonstrated in healthy volunteers that passive movement of a relaxed hand synchronized with voluntary movement of the other hand can effectively induce muscle activity in the former. This intervention leads the motor cortices to bilaterally active mode, and enhances their interhemispheric functional coupling. Further research is needed to determine how the putative sensory-motor association leads to the EMG increase. However, in the current work, not only the left (contralateral) but the right (ipsilateral) motor-cortical activity correlated with the EMG increase of the right relaxed hand (Figures 2C,D). The current work could not prove the causal relationship between these activities and the EMG increase through descending pathways. In primates, neurons in the ipsilateral motor cortex project to spinal interneurons (29, 30). Therefore, not only the contralateral but the ipsilateral activity might be directly involved in the EMG increase through the ipsilateral descending pathway. If so, the IN task could promote activity in this pathway [c.f. (31)], which could compensate hand motor function after contralateral stroke (32).

The current results were obtained from healthy young participants; therefore, the current intervention needs to be tested in stroke patients. There are several caveats to be considered when applying our intervention clinically. First, the patients must have intact proprioceptive pathways from the paralyzed side, since proprioceptive input (processing) from the paretic hand (most likely synchronized with the movement of the intact hand) could be crucial. However, even when proprioceptive pathways are damaged, viewing the movement of one’s own paretic hand synchronized with the voluntary movement of the intact hand might cause similar effects to bilateral mirror therapy (33–35). Second, complete damage to the motor cortex severely impairs both motor and proprioceptive processing (36). This study showed that a right-handed 71-year-old male patient with a focal subcortical hemorrhage over the left precentral hand region was unable to move his right arm/hand/fingers and could not experience proprioceptive illusory movement of the right hand in the third week after the stroke. (These functions were improved 6 months after stroke.) On the other hand, in the case of partial motor-cortical damage by an experimental ischemic block, the motor and proprioceptive functions seems to be compensated by spared adjacent tissue around stroke core (3). Hence, in the latter case, capability of residual tissue associating motor and proprioceptive signals would be the key.

We expect that the IN task may be effective in the acute and subacute phases of stroke when the brain spontaneously shifts to the bilaterally active mode (2). We assume that moving the immobile hand from the acute and subacute phases of stroke may decrease the risk of excessive interhemispheric inhibition from the contralesional (intact) motor cortex to the ipsilesional one, as shown in chronic phase (16). In addition, such early phase intervention might decrease the risk of spasticity or rigidity progression caused by long-term immobility of the paretic hand.

In the present paper, we focused on the IN task-related activity in the bilateral motor cortices. The reality, however, is that much broader sensory-motor cortical–subcortical networks are involved (Supplementary Figure 1). In addition to the bilateral motor cortices, the IN task-related activity (IN > LA + RP) was identified in the left secondary somatosensory cortex (SII), in the right area 2, intraparietal sulcus area (IPS), inferior parietal lobule (IPL), SII, ventral premotor cortex (PMv)/area 44, and in the left cerebellar hemisphere and vermis. The right inferior parieto-frontal cortices and the left cerebellar hemisphere and vermis are main constituents of proprioceptive processing network (23, 37). Hence, sensory-motor association during the IN task likely occurs not only in the bilateral motor cortices but also in the proprioceptive processing network. This means that a bilateral proprioceptive-motor coupling intervention allows for intervention not only in the bilateral motor cortices as previously thought, but also in the broader proprioceptive network. If one considers the fact that the PMv is capable of sending motor commands to the spinal cord (30) and of compensating motor function when the M1 is severely damaged (38, 39), proprioceptive-motor coupling in this region could be advantageous for recovery of hand motor function. Finally, one should bear in mind the possibility that the right inferior parieto-frontal and the left cerebellar damages may reduce the effectiveness of this intervention (40).

## 3 Improvement of hand dexterity utilizing left–right independent mode

The bilaterally active mode is accompanied by involuntary muscle activity and movement. After stroke, when the motor cortex ipsilateral to the hand is active, involuntary mirror movements and muscle activity of the opposite hand can occur (4, 41). Involuntary mirror movements and muscle activity can also be observed in children with immature interhemispheric inhibition between the left and right motor cortices and in older adults with reduced interhemispheric inhibition (42). Such involuntary movements are more likely to appear when the bilaterally active mode is overtrained. In addition, excessive activity in the motor cortex ipsilateral to the hand in children and older adults is closely related to their lower hand dexterity (24, 43). To circumvent this, training the motor cortex into a left–right independent mode, by training the left and right hands to perform different movements, may be effective. Indeed, the previous study has shown in healthy older adults that their left and right motor cortices become bilaterally active mode, probably due to aging-related decline of interhemispheric inhibition, but that 2-month bimanually different movement training can reactivate the inhibition and improve hand dexterity (24).

The bilaterally active mode of the left and right motor cortices observed in healthy older adults is similar to that observed after stroke. When the bilateral motor-cortical activation is a cause for the clumsiness of movement when a stroke patient becomes able to manage to move the paretic hand, training both hands to perform different movements could improve his/her interhemispheric inhibition and hand dexterity.

## 4 Conclusion

**Figure 3:**
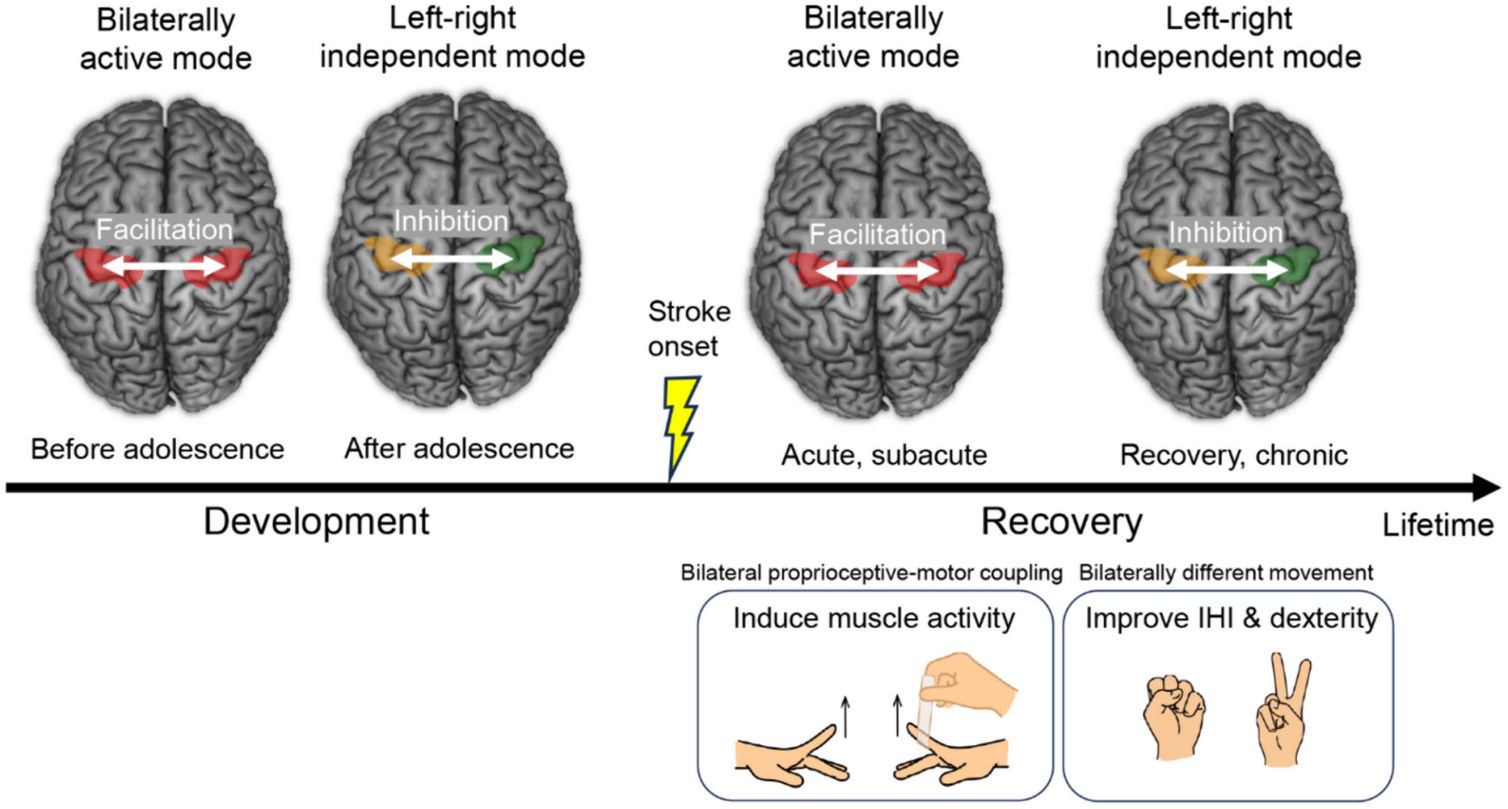
Transition of the left and right motor cortices from the bilaterally active mode to the left–right independent mode. This is a rule of typical development of hand motor function and also a common feature of motor function recovery after stroke. Proposed interventions during recovery are shown at right bottom. IHI, interhemispheric inhibition.

Our recent EMG and functional MRI study in healthy younger adults suggests that proprioceptive intervention of the paralyzed (immobile) hand synchronized with voluntary movement of the intact hand (bilateral proprioceptive-motor coupling intervention) could be worth applying for restoration of motor function of the paretic hand after stroke, utilizing the spontaneous bilaterally active mode of motor cortices frequently observed after stroke (Figure 3). This way, muscle activity could be induced in the paralyzed hand through the association between voluntary motor signals and proprioceptive inputs to the motor cortex. In addition, our previous functional MRI study in healthy older adults indicates the possibility that, when a stroke patient becomes able to manage to move the paretic hand but the movement is clumsy, training both hands to perform different movements (bilaterally different movement training) that guides the motor cortices into left–right independent mode could improve interhemispheric inhibition and hand dexterity (Figure 3). These are not a one-size-fits-all theory that can solve problems of all stroke patients (44). However, one could expect that the series of proposed interventions give a beneficial effect on hand motor functions of certain types of stroke patients.

The transition of the motor cortices from the bilaterally active mode to the left–right independent mode is a common feature of motor function recovery after stroke (2, 4), and also is a rule of development of hand motor function (Figure 3), which everyone has experienced during the developmental process (9, 10). The habilitation of “re-habilitation” means “to gain ability,” and it is during the developmental period that the most ability is gained in a person’s lifetime. Therefore, rehabilitation according to developmental rules may be the most natural and effective way for the brain to reacquire abilities. When planning rehabilitation strategies, taking advantage of the brain’s spontaneous recovery process and referring to its developmental rules may be important considerations.

## Data availability statement

The raw data supporting the conclusions of this article will be made available by the authors, without undue reservation.

## Ethics statement

The studies involving humans were approved by Minoru Tsukada, Ethics Committee of the National Institute of Information and Communications Technology. The studies were conducted in accordance with the local legislation and institutional requirements. The participants provided their written informed consent to participate in this study.

## Author contributions

HN: Conceptualization, Data curation, Formal analysis, Funding acquisition, Investigation, Methodology, Project administration, Resources, Supervision, Validation, Visualization, Writing – original draft, Writing – review & editing. YT: Conceptualization, Data curation, Formal analysis, Funding acquisition, Investigation, Methodology, Project administration, Resources, Supervision, Validation, Visualization, Writing – original draft, Writing – review & editing. TM: Conceptualization, Data curation, Formal analysis, Funding acquisition, Investigation, Methodology, Project administration, Resources, Supervision, Validation, Visualization, Writing – original draft, Writing – review & editing. EN: Conceptualization, Data curation, Formal analysis, Funding acquisition, Investigation, Methodology, Project administration, Resources, Supervision, Validation, Visualization, Writing – original draft, Writing – review & editing.
