# Supplementary material for: Theoretical proposal for restoration of hand motor function based on plasticity of motor-cortical interhemispheric interaction and its developmental rule
Source: Front Neurol. 2024 Jul 23;15:1408324. doi: 10.3389/fneur.2024.1408324 (PMC11304450; doi:10.3389/fneur.2024.1408324)
Supplement: Supplementary file 1 [file Data_Sheet_1.docx]

**Supplementary Material for**

Theoretical proposal for restoration of hand motor function utilizing plasticty of interhemispheric interaction between motor cortices

Hideki Nakano, Yandi Tang, Tomoyo Morita, Eiichi Naito

# **Supplementary methods**

## **Participants**

A total of 55 healthy right-handed young adults (37 men, 18 women: mean age, 21.9 [mean] ± 1.6 [standard deviation]; range, 19–26 years) participated in this study. None of them had a history of self-reported neurological, psychiatric, or motor disorders. Their handedness was confirmed using the Edinburgh handedness inventory (Oldfield, 1971). The study protocol was approved by the Ethics Committee of the National Institute of Information and Communications Technology and the MRI Safety Committee of the Center for Information and Neural Networks (CiNet; no. 2003260010). Details of the experiment were explained to each participant before the experiment, and they provided written informed consent. This study was conducted in accordance with the principles and guidelines of the Declaration of Helsinki (1975).

## **Motor tasks**

We prepared three motor tasks (Figure 1B). In a left finger active extension (left active; LA) task, the participants with their eyes closed extended their left index finger in synchronization with a computer-generated 1 Hz tone. They were asked to repeat 1 Hz extension and its following relaxation of the left finger, while relaxing their right hand. They were encouraged to extend the left finger to its maximum angle. In a right finger passive extension task (right passive; RP), experimenters extended their right relaxed index finger in synchronization with the 1 Hz tone. The blindfolded participants were asked to relax both hands, the experimenters (HN during EMG recoding and EN during fMRI experiment) repeated 1 Hz extension of the right finger to its maximum angle. In an in-phase (IN) task, the experimenters extended the right relaxed finger to its maximum angle (RP) at 1 Hz synchronized with the participant's active 1 Hz left finger extension (LA).

Before we started the tasks, we measured maximum extension angles of the left and right index fingers in each participant using an angle measuring device (easyangle, ITO PHYSIOTHERAPY&REHABILITATION Co., Ltd., Tokyo, Japan). The mean maximum angles across participants were 40.0 ± 8.0 and 40.4 ± 7.5 degrees for the left and right fingers, respectively.

All of the tasks were performed both during EMG recording and during fMRI experiment. The order of tasks (IN, RP, IN) was fixed for all participants. This way, when we evaluate inter-participant correlation between brain activity and EMG increase (Figure 2D), possible influence of task order difference across participants can be eliminated. In addition, we have confirmed in another experiment that the IN task alone (without being preceded by other (LA and RP) tasks) can generate the same effect (= EMG increase in the right relaxed hand), so that the effect observed during the IN task is not caused by accumulation of effects of preceding tasks. Both in the EMG recording and in the fMRI experiment, each participant completed one experimental run for each task. In a run, 20-s task epoch was repeated eight times with a 10-s rest phase in between. Each run also included a 10-s rest phase before the start of the first task epoch. The participants and experimenters were given computer-generated auditory instructions (“3, 2, 1, start” and “stop”) to inform them of the start and finish of a task epoch.

## **EMG recording and analysis**

EMG recording was conducted before the fMRI experiment outside the MRI scanner room. Each participant was seated in a chair. The left and right forearms were placed on a desk with towels laid on it, with their palms down. The mean distance between two hands across participants was 32.5 ± 6.3 cm.

In each task, surface electromyogram (EMG) was recorded from the right extensor digitorum communis (EDC). The skin surface was cleaned with gel (Nuprep, Nihonsanteku Co., Ltd., Osaka, Japan). Using double-sided adhesive tape (Map-3M402, Nihonsanteku Co., Ltd., Osaka, Japan), a bipolar Ag-AgCl surface electrode (BA-U410 m(A)-015, Nihonsanteku Co., Ltd., Osaka, Japan) was placed over the belly of the muscle. The EMG signals were recorded using the EMG Multi Analysis Programe MaP1038L (Nihonsanteku Co., Ltd., Osaka, Japan; sampling rate = 2048 Hz; high frequency filter = 1000 Hz; time constant = 0.03 sec). The EMG raw data were amplified (gain, 10000), and smoothed using a moving average.

During EMG recording, a care was taken in the environment to prevent noises from entering the recording. However, we were unable to completely eliminate background noise probably due to individual differences in electrode resistance in some participants. In addition, some participants showed small, occasional spontaneous firing of the extensor muscle even when their right hand was at rest. For these reasons, the EMG activity did not completely reach zero even during the rest phase (Figure 1B).

The root-mean-square EMG values from the first 2–19 s during the task epoch and from the first 2–7 s during the rest phase were calculated, and average values for the eight tasks and rest phases were calculated for each participant. For statistical analysis, a two-factorial analysis of variance (repeated measurement) for tasks (3) x period (2: task-rest) was performed (Figure 1B).

**fMRI experiment**

The participants were placed in the supine position in the MRI scanner, and performed the three motor tasks. The head was secured with a sponge cushion and adhesive tape, and the participants wore MRI-compatible earphones (SENSIMETRICS Model S14). The arms were set in a natural semi-rotated position, stretched along the body and relaxed. Both the left and right hands were placed on hand cushions, and relaxed with their palms down and fingers naturally stretched. They were instructed to close their eyes, relax their entire bodies without producing unnecessary movements, and think only of things relevant to the tasks assigned. We told them to close their eyes just before each experimental run.

**fMRI acquisition**

Functional images were acquired using T2*-weighted gradient echo-planar imaging (EPI) sequences on a 3.0-Tesla MRI scanner (Vida; Siemens, Germany) equipped with a 64-channel array head-neck coil. A multiband imaging technique was used (multiband factor, 3; Moeller et al. 2010). Each volume consisted of 51 slices (slice thickness, 3.0 mm with no inter-slice thickness) acquired in an interleaved manner, covering the entire brain. The time interval between successive acquisitions from the same slice (TR) was 1,000 ms. An echo time (TE) of 30 ms and a flip angle (FA) of 60° were used. The field of view (FOV) was 210 x 210 mm^2^ and the matrix size was 70 x 70 pixels. The voxel dimensions were 3 × 3 × 3 mm^3^ in the x-, y-, and z-axes, respectively. Each participant completed one 250-s experimental run for each task (see above), which included a 10-s period (before the first rest phase) that was excluded from the analysis to eliminate the influence of unsteady magnetization.

Prior to EPI acquisition, filed map images were obtained using a gradient echo sequence (TR = 1000 ms, TE1 = 5.29 ms or TE2 = 7.75 ms, FOV = 210 x 210 mm^2^, FA = 60°, matrix size = 70 x 70 pixels, number of slices = 51, voxel dimensions = 3 × 3 × 3 mm^3^), in order to correct geometrical distortion of EPI images caused by static field inhomogeneities (see below).

As an anatomical reference, a T1-weighted magnetization-prepared rapid gradient echo (MP-RAGE) image was acquired using the same scanner. The imaging parameters were as follows: TR = 1900ms, TE = 2.48ms, FA = 9°, FOV = 256 × 256 mm^2^, matrix size = 256 × 256 pixels, slice thickness = 1.0 mm, voxel size = 1 × 1 × 1 mm^3^, and 208 contiguous sagittal slices.

**fMRI analysis**

**Preprocessing**

To eliminate the influence of unsteady magnetization during the tasks, the first 10 volumes (10 second) of EPI images in each run were excluded from the analysis. Acquired imaging data were analyzed using SPM 12 (default setting: Wellcome Trust Centre for Neuroimaging, London, UK) running on MATLAB R2017a (MathWorks, Sherborn, MA, USA).

A voxel-displacement map (VDM) was calculated from the acquired field map images. EPI images were aligned to the first image (realigned images). Time series data of the head position during the fMRI experiment were obtained by a rigid body transformation (linear transformation) using the least squares method for six realign parameters (translation along the x-, y-, and z-axes and the rotational displacements of pitch, raw, and roll). Then, the head movements of the participants were evaluated by the framewise displacement (FD) values based on the six parameters (Power et al., 2012). To inspect FD values through all frames of an entire experimental run, we counted the number of frames that had an FD of over 0.9 mm in each participant according to a previous study (Siegel et al., 2014). None of the participants showed FD values that exceeded 0.9 mm in more than 5 % of the total volumes, and thus none of them were excluded from the following analyses.

The realigned images were unwarped using the VDM to correct geometrical distortion of these images. In each participant, the T1-weighted structural image was co-registered to the mean image of all realigned and unwarped EPI images. The individual co-registered T1-weighted structural image was spatially normalized to the standard stereotactic Montreal Neurological Institute (MNI) space (Evans et al., 1994). Applying the parameter estimated in this process, the individual realigned and unwarped images were normalized to the MNI space with 2-mm isotropic voxel size using the SPM12 normalization algorithm. Finally, the normalized images were filtered using a Gaussian kernel with a full-width at half-maximum (FWHM) of 4 mm along the x-, y-, and z-axes.

**Single-subject analysis**

After preprocessing, we first explored task-related activations and deactivations in each participant with a general linear model (Friston et al., 1995; Worsley & Friston, 1995). This was done for each task. For the first-level analysis, a design matrix was prepared for each participant. The design matrix contained a boxcar function for the task epoch that was convolved with a canonical hemodynamic response function (HRF). Six realignment parameters were also included in the design matrix as regressors to correct for residual motion-related noise after the realignment. Contrast images showing activation (task > rest) and deactivation (rest > task) in each task and the image of IN vs. LA + RP were created for each participant, which was used in the following second-level group analysis. In the first-level analysis, global mean scaling was not performed to avoid inducing type I error in the assessment of negative blood oxygenation-level dependent (BOLD) responses (Aguirre et al., 1998).

**Group analysis**

In the second-level group analysis, we first examined activations and deactivations in each task (not shown in Figure). Next, we examined if the bilateral motor cortices are active during the IN task when compared with the LA and RP tasks (IN > LA + RP). In these analyses, we generated a voxel-cluster image using an uncorrected voxel-wise threshold of p < 0.005, and evaluated the significance of brain activations in terms of the spatial extent of the activations in the entire brain (p < 0.05, family-wise-error (FWE) corrected). Since our main regions-of-interest were the bilateral motor cortices (dorsal premotor cortex and primary motor cortex), the present study only reported motor-cortical activations (Figure 2A red sections). For anatomical identification of the activation peaks, we referred to the cytoarchitectonic probability maps (Eickhoff et al., 2005; Amunts et al., 2020).

In order to visualize task-dependent activity change in the identified bilateral motor-cortical clusters (Figure 2A red sections), we extracted individual brain activity (parameter estimates) from the peaks of left (x, y, z = -38, -16, 50) and right (36, -16, 52) motor-cortical clusters for each task, and calculated the mean across participants (Figure 2B). Finally, we also show the IN task-related activity (IN > LA + RP) in the entire brain (see Supplementary results and Figure 1).

**Task-related functional connectivity analysis**

We further examined the brain regions where activity enhanced functional coupling with the activity in the left or right motor-cortical cluster (seed regions; Figure 2A red) during the IN task when compared to the other (LA and RP) tasks, by conducting a generalized psychophysiological interaction analysis (gPPI) (McLaren et al., 2012). Defining seed regions from the contrast analysis and using the seed regions in the functional connectivity analysis is completely different from the circular analysis where a ROI is defined by an identified cluster in one contrast of activation analysis and then used for another contrast in the same analysis (Wei et al., 2022). This analysis was performed on SPM-preprocessed fMRI data using the CONN toolbox version 20.b (Whitfield-Gabrieli & Nieto-Castanon, 2012; Nieto-Castanon, 2020). Physiological noises originating from the white matter and cerebrospinal fluid were removed using the component-based noise correction method (CompCor) in the toolbox (Behzadi et al., 2007). Head motion-related artifacts, scrubbing, and condition effects were also removed. A temporal band-pass filter of 0.008–0.09 Hz was applied, because we wanted to examine task-related functional connectivity change in this slower range of brain activity fluctuation below than the cardiac and respiratory cycles (0.1–1.2 Hz) (Cordes et al. 2001).

In the gPPI analysis, we used each of the left and right motor-cortical clusters as a seed region. In each participant, the time course of the average fMRI signal across the voxels in each seed region was deconvolved using the canonical HRF (physiological variable). Then, we performed a general linear model analysis using the design matrix and included the following regressors: physiological variable, boxcar function for the task epoch (psychological variable), and multiplication of the physiological variable and the psychological variable (PPI). These variables were convolved with a canonical HRF. Six realignment parameters were also included in the design matrix as regressors of no interest.

In each task, we first generated an image of voxels showing to what extent their activities changed with the PPI regressor of each seed region in each participant. Then, we generated a contrast image (IN > LA + RP) that shows the IN task-related connectivity change for each participant. We used this individual image in the second-level group analysis. In the second-level analysis, we searched for significant clusters in the entire brain (uncorrected voxel-wise threshold of p < 0.005 and extent threshold of p < 0.05 FWE-corrected). We only reported the clusters identified around the bilateral motor cortices (Figure 2A orange) in this study.

**Correlation analysis**

Finally, we examined if the IN task-related motor-cortical activity (IN > LA + RP) correlates with the EMG increase (task > rest) in the right extensor muscles during the IN task across participants. A correlation analysis was conducted using individual EMG activity measured outside MRI as a covariate. Since 44 of 55 participants showed EMG increase during the task epoch compared to the rest phase during the IN task, the correlation analysis was done for the 44 participants. The individual raw data of EMG increase were not normally distributed, therefore, the logarithm of the data was calculated and used as covariates. We first generated a cluster image using uncorrected voxel-wise threshold of p < 0.005. Since our main interest was the bilateral motor cortices, in the statistical evaluation, we conducted small volume correction (SVC; p < 0.05 FWE-corrected) approach, using a sphere with 8 mm radius around the peak (-38, -16, 50) of the IN task-related left M1 activity or a sphere with 8 mm radius around the peak (36, -16, 52) of the IN task-related right M1 activity respectively (Figure 2C). The 8 mm radius was selected based on final smoothness of functional image (maximum 7.1 mm FWHM). We extracted individual brain activity (parameter estimates) from a peak of each identified cluster (-34, -14, 48 for the left and 40, -14, 52 for the right), and displayed interparticipant correlation between the brain activity and the logarithm of EMG activity (Figure 2D).

# **Supplementary results and discussion**

In the present paper, we focused on the IN task-related activity in the bilateral motor cortices. The reality, however, is that much broader sensory-motor cortical-subcortical networks are involved (Supplementary Figure 1). In addition to the bilateral motor cortices, the IN task-related activity (IN > LA + RP) was identified in the left secondary somatosensory cortex (SII), in the right area 2, intraparietal sulcus area (IPS), inferior parietal lobule (IPL), SII, ventral premotor cortex (PMv)/area 44, and in the left cerebellar hemisphere and vermis. The right inferior parieto-frontal cortices and the left cerebellar hemisphere and vermis are main constituents of proprioceptive processing network (Naito et al., 2016; Morita and Naito, 2022). Hence, sensory-motor association during the IN task likely occurs not only in the bilateral motor cortices but also in the proprioceptive processing network. This means that a bilateral proprioceptive-motor coupling intervention allows for intervention not only in the bilateral motor cortices as previously thought, but also in the broader proprioceptive network. If one considers the fact that the PMv is capable of sending motor commands to the spinal cord (Morecraft et al., 2019) and of compensating motor function when the M1 is severely damaged (Nudo, 2006; Yamamoto et al., 2019), proprioceptive-motor coupling in this region could be advantageous for recovery of hand motor function. Finally, one should bear in mind the possibility that the right inferior parieto-frontal and the left cerebellar damages may reduce the effectiveness of this intervention (Chilvers et al., 2022).

**Supplementary references**

Aguirre, G. K., Zarahn, E., and Esposito, M. D. (1998). The inferential impact of global signal covariates in functional neuroimaging analyses. Neuroimage 8, 302–306. doi: 10.1006/nimg.1998.0367.

Amunts, K., Mohlberg, H., Bludau, S., and Zilles, K. (2020). Julich-Brain: A 3D probabilistic atlas of the human brain’s cytoarchitecture. Science 369(6506), 988–992. doi:10.1126/science.abb4588.

Behzadi, Y., Restom, K., Liau, J., and Liu, T. T. (2007). A component based noise correction method (CompCor) for BOLD and perfusion based fMRI. NeuroImage 37(1), 90–101. doi:10.1016/j.neuroimage.2007.04.042.

Chilvers, M. J., Low, T. A., and Dukelow, S. P. (2022). Beyond the dorsal column medial lemniscus in proprioception and stroke: A white matter investigation. Brain Sci 12, 1651. doi.org/10.3390/brainsci12121651.

Cordes, D., Haughton, V. M., Arfanakis, K., Carew, J. D., Turski, P. A., Moritz, C. H. et al. (2001). Frequencies contributing to functional connectivity in the cerebral cortex in “resting-state” data. Am J Neuroradiol 22, 1326–1333.

Eickhoff, S. B., Stephan, K. E., Mohlberg, H., Grefkes, C., Fink, G. R., Amunts, K. et al. (2005). A new SPM toolbox for combining probabilistic cytoarchitectonic maps and functional imaging data. Neuroimage 25, 1325–1335. doi:10.1016/j.neuroimage.2004.12.034.

Evans, A. C., Kamber, M., Collins, D. L., and Macdonald, D. (1994). An mri-based probabilistic atlas of neuroanatomy. Magnetic resonance scanning and epilepsy 263–274.

Friston, K. J., Holmes, A. P., Poline, J. B., Grasby, P. J., Williams, S. C. R., Frackowiak, R. S. et al. (1995). Analysis of fMRI time-series revisited. Neuroimage 2(1), 45–53. [doi:10.1006/nimg.1995.1007](https://doi.org/10.1006/nimg.1995.1007).

Mclaren, D. G., Ries, M. L., Xu, G., and Johnson, S. C. (2012). A generalized form of context-dependent psychophysiological interactions (gPPI): A comparison to standard approaches. NeuroImage 61(4), 1277–1286. doi:10.1016/j.neuroimage.2012.03.068.

Moeller, S., Yacoub, E., Olman, C.A., Auerbach, E., Strupp, J., Harel, N. et al. (2010). Multiband multislice GE-EPI at 7 tesla, with 16-fold acceleration using partial parallel imaging with application to high spatial and temporal whole-brain fMRI. Magnetic resonance in medicine 63, 1144–1153. doi:10.1002/mrm.22361.

Morecraft, R. J., Ge, J., Stilwell-Morecraft, K. S., Rotella, D. L., Pizzimenti, M. A., and Darling, W. G. (2019). Terminal organization of the corticospinal projection from the lateral premotor cortex to the cervical enlargement (C5-T1) in rhesus monkey. J Comp Neurol 527, 2761–2789. doi:[10.1002/cne.24706](https://doi.org/10.1002/cne.24706).

Morita, T., and Naito, E. (2022). Facilitation of hand proprioception processing in paraplegic individuals with long-term wheelchair sports training. Brain Sci 22, 1295. doi.org/10.3390/brainsci12101295.

Naito, E., Morita, T., and Amemiya, K. (2016). Body representations in the human brain revealed by kinesthetic illusions and their essential contributions to motor control and corporeal awareness. Neurosci Res 104, 16–30. doi:[10.1016/j.neures.2015.10.013](https://doi.org/10.1016/j.neures.2015.10.013).

Nieto-Castanon, A. (2020). Handbook of functional connectivity Magnetic Resonance Imaging methods in CONN. In Handbook of functional connectivity Magnetic Resonance Imaging methods in CONN (Issue February). doi:10.56441/hilbertpress.2207.6598.

Nudo, R. J. (2006). Mechanisms for recovery of motor function following cortical damage. Curr Opin Neurobiol 16, 638–644. doi:[10.1016/j.conb.2006.10.004](https://doi.org/10.1016/s1047-9651(02)00054-2).

Oldfield, R. C. (1971). The assessment and analysis of handedness: the Edinburgh inventory. Neuropsychologia 9(1), 97–113.

Power, J. D., Barnes, K. A., Snyder, A. Z., Schlaggar, B. L., and Petersen, S. E. (2012). Spurious but systematic correlations in functional connectivity MRI networks arise from subject motion. NeuroImage 59(3), 2142–2154. doi:10.1016/j.neuroimage.2011.10.018.

Siegel, J. S., Power, J. D., Dubis, J. W., Vogel, A. C., Church, J. A., Schlaggar, B. L. et al. (2014). Statistical improvements in functional magnetic resonance imaging analyses produced by censoring high-motion data points. Human Brain Mapping 35(5), 1981–1996. doi:10.1002/hbm.22307.

Wei, P., Bao, R., and Fan, Y. (2022). Independence of functional connectivity analysis in fMRI research does not rely on whether seeds are exogenous or endogenous. Medicine in Novel Technology and Devices 15, 100126. doi:10.1016/j.medntd.2022.100126.

Whitfield-Gabrieli, S., and Nieto-Castanon, A. (2012). Conn : A Functional Connectivity Toolbox for Correlated and Anticorrelated Brain Networks. Brain connectivity 2(3), 125–141. doi:10.1089/brain.2012.0073.

Worsley, K. J., and Friston, K. J. (1995). Analysis of fMRI time-series revisited-again. Neuroimage 2(3), 173–181. doi:10. 1006/nimg.1995.1023.

Yamamoto, T., Hayashi, T., Murata, Y., Ose, T., Higo, N. (2019). Premotor cortico-cerebellar reorganization in a macaque model of primary motor cortical lesion and recovery. J Neurosci. 39, 8484–8496. doi:10.1523/JNEUROSCI.0077-19.2019.


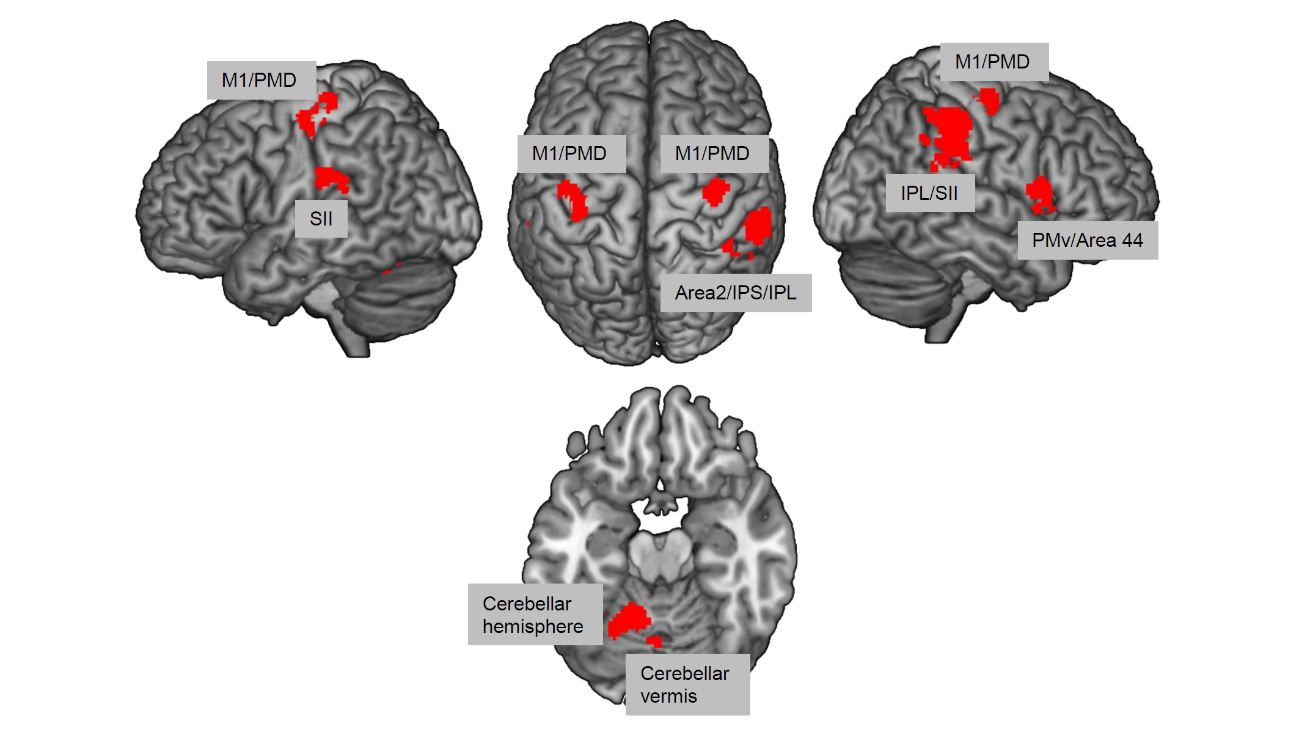


Supplementary Figure 1 IN task-related activity in the entire brain. In addition to the bilateral motor cortices, the IN task-related activity (IN > LA + RP) was identified in the left secondary somatosensory cortex (SII), in the right area 2, intraparietal sulcus area (IPS), inferior parietal lobule (IPL), SII, ventral premotor cortex (PMv)/area 44, and in the left cerebellar hemisphere and vermis. The activities are superimposed on the standard brain of the Montreal Neurological Institute. The right inferior parieto-frontal cortices and the left cerebellar hemisphere and vermis are main constituents of proprioceptive processing network. The results suggest that proprioceptive-motor coupling may also occur in this network during the IN task, in addition to the bilateral motor cortices.
